# Advanced glycation end products cause RAGE‐dependent annulus fibrosus collagen disruption and loss identified using in situ second harmonic generation imaging in mice intervertebral disk in vivo and in organ culture models

**DOI:** 10.1002/jsp2.1126

**Published:** 2020-09-21

**Authors:** Robert C. Hoy, Danielle N. D'Erminio, Divya Krishnamoorthy, Devorah M. Natelson, Damien M. Laudier, Svenja Illien‐Jünger, James C. Iatridis

**Affiliations:** ^1^ Leni & Peter W. May Department of Orthopaedics Icahn School of Medicine at Mount Sinai NY United States USA; ^2^ Department of Orthopaedics Emory University School of Medicine Atlanta Georgia USA

**Keywords:** advanced glycation end products, collagen degradation, collagen hybridizing peptide, intervertebral disk degeneration, second harmonic generation, texture analysis

## Abstract

Aging and diabetes are associated with increased low‐back pain and intervertebral disk (IVD) degeneration yet causal mechanisms remain uncertain. Advanced glycation end products (AGEs), which accumulate in IVDs from aging and are implicated in diabetes‐related disorders, alter collagen and induce proinflammatory conditions. A need exists for methods that assess IVD collagen quality and degradation in order to better characterize specific structural changes in IVDs due to AGE accumulation and to identify roles for the receptor for AGEs (RAGE). We used multiphoton microscopy with second harmonic generation (SHG), collagen‐hybridizing peptide (CHP), and image analysis methods to characterize effects of AGEs and RAGE on collagen quality and quantity in IVD annulus fibrosus (AF). First, we used SHG imaging on thin sections with an in vivo dietary mouse model and determined that high‐AGE (H‐AGE) diets increased AF fibril disruption and collagen degradation resulting in decreased total collagen content, suggesting an early degenerative cascade. Next, we used in situ SHG imaging with an ex vivo IVD organ culture model of AGE challenge on wild type and RAGE‐knockout (RAGE‐KO) mice and determined that early degenerative changes to collagen quality and degradation were RAGE dependent. We conclude that AGE accumulation leads to RAGE‐dependent collagen disruption in the AF and can initiate molecular and tissue level collagen disruption. Furthermore, SHG and CHP analyzes were sensitive to collagenous alterations at multiple hierarchical levels due to AGE and may be useful in identifying additional contributors to collagen damage in IVD degeneration processes.

## Graphical abstract

Multiphoton microscopy with second harmonic generation (SHG), collagen‐hybridizing peptide (CHP), and image processing analyses were used to characterize effects of advanced glycation end products (AGEs) on collagen quality and quantity in intervertebral disc annulus fibrosus (AF). High‐AGE diets increased AF fibril disruption and collagen degradation resulting in collagen loss in an in vivo dietary mouse model. An ex vivo IVD organ culture model of AGE challenge on wild type and receptor for AGE‐knockout (RAGE‐KO) mice with novel in situ SHG imaging determined this collagen degradation was RAGE dependent.

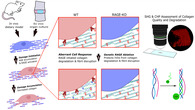

## 1 INTRODUCTION

Back pain is a leading cause of global disability commonly associated with intervertebral disk (IVD) degeneration.^1^ IVD degeneration is an aberrant, cell‐mediated response to progressive structural failure.^2^ The relationship between back pain and IVD degeneration is complex, resulting in an etiology that is multifactorial and heterogeneous, highlighting a need to understand structural changes and biological responses. Aging and diabetes are identified as risk factors associated with increased IVD degeneration and back pain.^3^, ^4^, ^5^, ^6^ These associations may be attributed to chronic proinflammatory conditions, yet these associations are confounded by environmental and genetic factors, making causal relationships difficult to identify.^7^, ^8^, ^9^ A leading hypothesis for a relationship between diabetes and IVD degeneration is the formation and accumulation of advanced glycation end products (AGEs) in diabetic IVD tissue.^10^, ^11^ AGEs are highly oxidant compounds that accumulate in aging and are implicated in diabetic complications that are known to cause structural and biological alterations to collagen and the extracellular matrix (ECM).^12^

There is mounting evidence for a causal relationship between IVD degeneration and AGEs. AGEs can accumulate in spinal tissues from aging, high‐AGE (H‐AGE) diets (eg, highly processed western diets) and diabetes, and are associated with structural changes in the IVD including decreased glycosaminoglycan content,^13^, ^14^, ^15^ increased vertebral bone changes,^16^ and increased collagen degradation.^15^ In addition, the receptor for AGEs (RAGE) has been observed to initiate an NF‐kB mediated inflammatory response in both human and mice IVD tissue exposed to AGEs.^17^, ^18^

The specific structural changes to the IVD ECM due to AGE exposure in the presence of RAGE are not well‐understood and we believe this is partly due to limitations in the methods used to identify early degenerative changes to the ECM that mark the initiation of a degenerative cascade. Recently, we demonstrated that dietary accumulation of AGEs in the IVD increased levels of molecular level collagen degradation,^15^ highlighting the direct contributions that AGEs can make to IVD degeneration. We believe that an improved understanding of the factors that cause early degenerative changes and the events that are subsequent to initiation of these changes may inform methods to detect and prevent such contributors to IVD degeneration. This motivates the development of tools to characterize changes in collagen quality and quantity in response to AGEs, as well as an investigation into the role of RAGE in initiating these structural changes.

Multiphoton microscopy is a powerful tool to analyze the ECM of collagenous tissues due to the emission of the second harmonic generation (SHG) signal from fibrillar collagen. The intensity of the SHG signal is related to several biological characteristics of collagen including collagen orientation,^19^, ^20^ collagen fibril diameter,^21^ total collagen content,^22^, ^23^ collagen degradation,^24^, ^25^ and collagen crosslinking.^26^, ^27^ In addition to the quantification of mean SHG intensity, second order statistics (eg, entropy, energy, and inertia) of SHG images proved useful in characterizing collagen structure in tendon, cardiac, and tumor tissues.^28^, ^29^, ^30^, ^31^, ^32^ Percent area calculations of positive SHG pixels above a threshold value has been used extensively to assess the total amount of collagen.^33^, ^34^, ^35^, ^36^ SHG is particularly amenable to investigations of the annulus fibrosus (AF) in the IVD since the AF is abundant in collagen type I. We believe that the development of these image analysis tools for application assessing collagen quality and quantity in the AF will provide methods that improve understanding of collagen changes in early IVD degeneration and also increase understanding of ECM changes in response to AGE exposure and accumulation.

**FIGURE 1:**
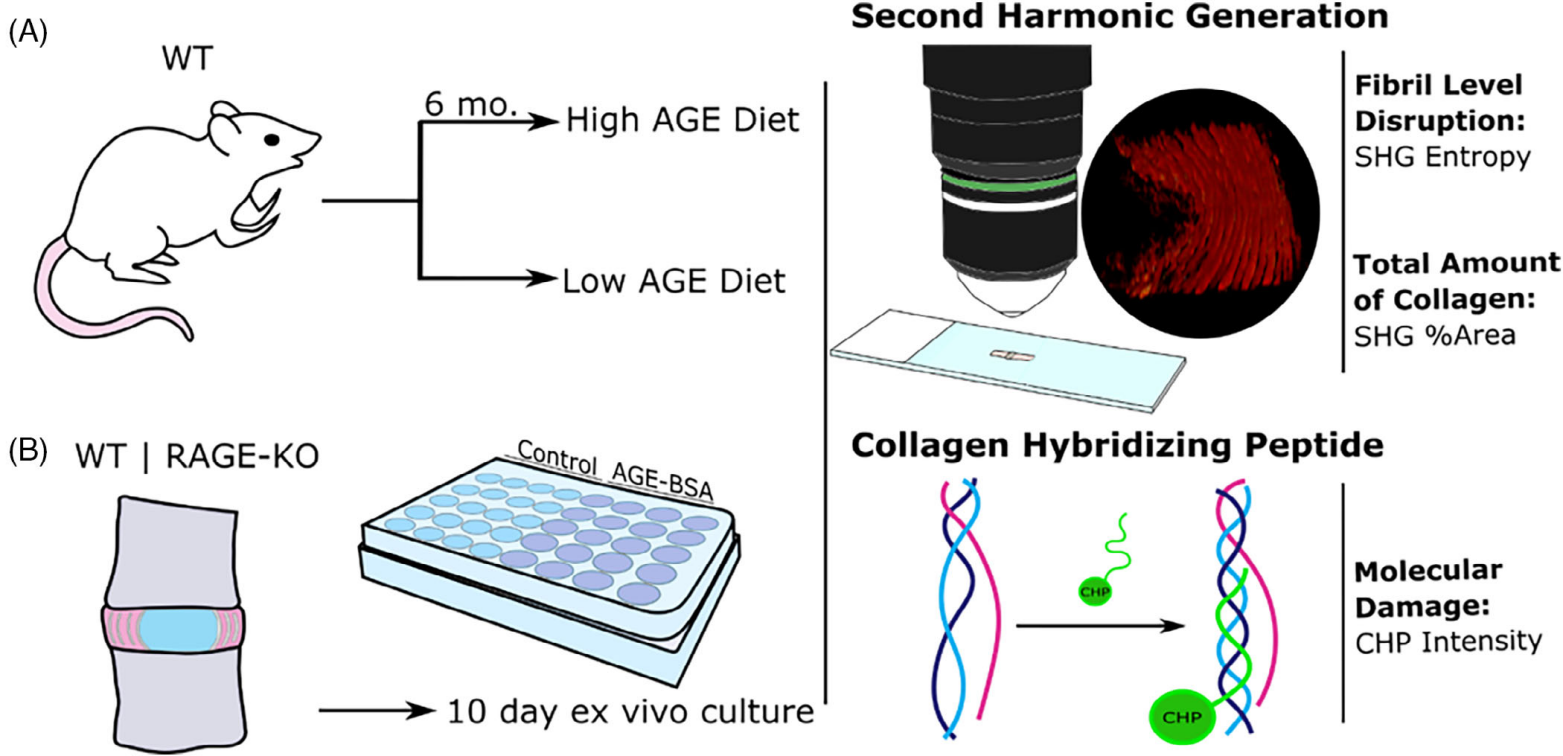
Study design. A, Dietary mouse model and B, ex vivo organ culture was used to assess the role of AGEs and RAGE in the initiation of early degenerative changes in the IVD. In situ SHG imaging was performed to assess collagen structure and amount. CHP was performed to assess molecular collagen damage. AGEs, Advanced glycation end products; CHP, collagen‐hybridizing peptide; IVD, intervertebral disk; RAGE, receptor for AGEs; RAGE‐KO, RAGE‐knockout; SHG, second harmonic generation; WT, wildtype

This two‐part study used in vivo and ex vivo IVD model systems with wild type and RAGE‐knockout (RAGE‐KO) mice in order to investigate changes in AF collagen quality and degradation in response to AGE challenges using SHG methods (Figure 1). We first hypothesized that loss of SHG intensity and collagen degradation due to H‐AGE diet will result in AF collagen fibril disruption and a loss of overall collagen. To test this hypothesis we utilized a dietary mouse model, validated previous SHG Intensity and collagen‐hybridizing peptide (CHP) results,^15^ and performed new SHG image analysis to measure changes to fibril disruption and collagen quantity. The second hypothesis was that this early degenerative cascade is a result of resident IVD cell activity via the recognition of AGE ligands through RAGE, leading to degradation of the surrounding collagen. We isolated cell‐mediated effects from biomechanical or systemic level effects by using an ex vivo organ culture model to evaluate the effects of AGEs and RAGE in WT and RAGE‐KO mice. Using the organ culture model system, we could assess the role of IVD cells in early degenerative collagenous changes in the absence of systemic alterations (eg, hormonal, immune and/or biomechanical) to the animal from AGEs.^37^ Collagen degradation, quality, and quantity were observed using CHP and SHG imaging methods and quantitative image analysis using intensity and morphometric approaches to measure differences between groups. Furthermore, SHG analyzes were performed with thin sections for the in vivo model and with in situ imaging in the organ culture to determine if both methods can characterize ECM alterations, and to provide a proof‐of‐concept for the potential of nondestructive situ imaging with SHG.

## 2 METHODS

### 2.1 Study design

Aim 1 characterized collagen quality and quantity in the AF of IVD tissues from mice fed either a high or low‐AGE diet for 6 months. Aim 2 characterized collagen quality and quantity in the AF of IVD tissues using an ex vivo organ culture model of both WT and RAGE‐KO IVDs. Specimens were analyzed with SHG and CHP and image processing algorithms to assess mean SHG intensity, SHG entropy as a measure of collagen fiber disruption, percent SHG area fraction as a measure of collagen quantity, and CHP intensity as a measure of collagen damage. Aim 1 used thin sections for SHG and CHP analyzes. Aim 2 used in situ SHG analyzes and used thin sections for CHP analyzes. Further, Aim 1 used animals of both sexes, and since sex had no significant effect for AF parameters we pooled for sex. Due to the lack of a significant sex effect in the AF parameters a single sex (males) was selected in Aim 2 studies to minimize variance and size differences.

### 2.2 In vivo and ex vivo organ culture mouse model

#### 2.2.1 In vivo model dietary mouse model

All experiments were performed in accordance with the Mount Sinai Institutional Animal Care and Use Committee. Mice from a previous study^15^ were used to assess the effect of dietary AGE ingestion on AF collagen fibril structure and quantity in vivo. A mixed population of 23 male and female wildtype (WT) C57BL/6J mice were each assigned to two isocaloric diet groups after weaning, and received either a low‐AGE chow (L‐AGE, n = 12) containing 7.6 μg/mg AGE (Test Diet Low AGE 5053; WF Fisher & Son CO, New Jersey) or high‐AGE (H‐AGE, n = 11) chow containing 40.9 μg/mg AGE generated via high‐temperature heating (NIH‐31 open formula chow autoclaved for 30 minutes at 120°C). Mice were euthanized at 6 months of age by cardiac puncture. Following sacrifice, Lumbar (L) L3‐L4 and L4‐L5 segments were dissected and fixed in 10% neutral buffered formalin prior to poly methyl methacrylate (PMMA) resin embedding for histological multiphoton and CHP analysis, following embedding procedures previously described.^38^ H‐AGE diet did not cause obesity or hyperglycemia in males or females, as previously reported.^15^

#### 2.2.2 Ex vivo organ culture mouse model

Five male WT C57BL/6J and five male RAGE‐KO mice were euthanized at 4 to 6 months by CO_2_ overdose and bathed in 70% ethanol for 2 minutes before dissection. Four caudal IVDs from coccygeal levels 2 to 5 and half of the adjacent superior and inferior vertebrae were harvested from each WT and RAGE‐KO mouse and immediately washed with primocin and penicillin streptomycin. Two levels were randomly separated into a control group and the remaining two were placed in the AGE‐bovine serum albumin (AGE‐BSA; ab51995; Abcam) group. Each level was placed in a 24 well plate, and cultured for 10 days in control or AGE‐BSA supplemented media. Control media included Dulbecco's modified Eagle's medium: Nutrient mixture (DMEM; Gibco; Carlsbad, California), 10% fetal bovine serum (FBS), 0.2% ascorbic acid, 1% penicillin streptomycin, 0.2% primocin, and 20 μg/mL BSA while AGE‐BSA supplemented media was identical save BSA was replaced at the same concentration with AGE‐BSA. Previously published measurements showed a difference of serum AGE concentration of 22.2 μg/mL between H‐LAGE and L‐AGE female mice. The concentration of 20 μg/mL was therefore chosen to represent the physiological difference to model ex vivo.^15^ Media was exchanged daily and the culture plate was kept on a rocker in an incubator at 37°C, 5% CO_2_, 5% O_2_, and > 90% humidity. All IVDs underwent a 24 hour preconditioning period in control media, after which the experimental group received AGE‐BSA supplemented media. Following the culture period, a subset of IVDs each were immediately fixed in 4% formaldehyde, resin embedded, and sectioned for CHP assessment. The remaining IVDs were flash frozen in liquid N_2_ and stored in −80°C until in situ multiphoton imaging. Similar organ culture procedures were previously applied to mice IVDs in our lab demonstrating metabolically active cells and no evidence for apoptosis.^39^ Cell viability was validated in this study using double staining of thiazolyl blue tetrazolium bromide (MTT, Sigma‐Aldrich, St. Louis, Missouri) to stain metabolically active cells and 4′,6‐diamidino‐2‐phenylindole (DAPI, Roche Diagnostics, Germany) to stain cell nuclei, as described.^40^ Live cells were confirmed on a single motion segment by the presence of dual staining robust MTT staining that aligned closely with DAPI staining (Supplementary Figure 1).

#### 2.2.3 In situ multiphoton microscopy

Prior to imaging, whole IVDs from organ culture were washed in dd‐H2O and halved using a size 10 surgical scalpel along the mid‐sagittal plane. In situ imaging was performed on an Olympus FV1000 MPE laser‐scanning microscope (Olympus Corporation). Optical slices of the anterior AF were taken through a depth of 100 μm at a step size of 10 μm. The anterior AF provided the largest viewing surface of collagenous tissue in the mid‐sagittal plane. The anterior AF was identified through a combination of larger size relative to the posterior AF, and tissue landmarks on the posterior side of the segments. Multiphoton excitation was performed with the tunable Coherent Chameleon Vision II laser. Backward SHG (b‐SHG) signal propagation was collected using the dedicated Olympus XLPLN water immersive 25X objective with a numerical aperture of 1.05. Excitation for b‐SHG was performed at 910 nm and recorded by a PMT at 440 +/− 20 nm. All parameters (ie, laser intensity, gain, high voltage, dwell time, and aspect ratio) were selected to minimize background noise while maximizing signal distribution without over‐saturation on the first sample of the series. These parameters were held constant for subsequent in situ imaging to allow comparison of image intensity across samples.

#### 2.2.4 Histological multiphoton microscopy

PMMA embedded IVDs from the in vivo dietary mouse model were sectioned mid‐sagittally at 5 μm and used for multiphoton microscopy. The multiphoton setup was used as described above and optical sections with a step size of 1.5 μm were taken through a depth of ~10 μm.

### 2.3 Image analysis and quantification

Intensity and morphometric evaluation was performed using a custom Matlab (The Mathworks Inc., Natick, Massachusetts) code to quantify the SHG intensity, percent area, and entropy of collagen in the IVD. Background subtraction was first performed on SHG images and a 500 x 500 pixel^2^ region of interest in the central anterior AF was selected for evaluation. The mean intensity of pixels within this region of interest was measured for comparison among the groups. To assess changes in the morphology of collagen structure independent of changes in intensity, a histogram equalization was performed and the entropy of the images was measured to determine the complexity of the collagen structures. Lower entropy values indicate a loss in the complexity of the AF structure, indicating a disruption to normal fibril and fiber architecture. These images were then binarized with the built in Otsu segmentation function to determine SHG signal above an automated threshold. The percent area of positive pixels was calculated as a measure of percent area of collagen and compared among groups.

### 2.4 Collagen hybridizing peptide

To assess IVDs for molecular damage to collagen, PMMA‐embedded mid‐sagittal lumbar sections were stained using collagen hybridizing peptide‐biotin conjugate (B‐CHP; BIO300, 3Helix Inc; Salt Lake City, Utah) or fluorescein conjugate (F‐CHP; FLU600, 3Helix Inc; Salt Lake City, Utah) to detect the presence of collagen damage as described previously.^24^, ^25^, ^41^ This procedure includes the creation of a 2 μM CHP solution, heating of the solution at 80°C for 5 minutes to monomerize the peptides, quenching in an ice bath for 30 seconds, and application of the solution to the tissue. The tissue was incubated overnight, and then positive binding of B‐CHP was detected using green fluorescent protein‐labeled streptavidin (Dylight 488 Strepavidin; Vector Laboratories Inc., Burlingame, California). F‐CHP was immediately imaged using a Leica DM6 widefield microscope with a FITC filter cube. To create a positive control for collagen damage, we subjected IVD motion segments to heat treatment, as previously described.^24^, ^42^ Caudal functional spine units from 6‐month old mice were dissected, stored in −80°C. Caudal motion segments were then thawed, placed in histology cassettes in a glass beaker with distilled water and subject to 90°C for 120 minutes. Heated spine units and their unheated control levels were then fixed in 4% formalin and embedded in PMMA for SHG and CHP assessments (Supplementary Figure 2).

### 2.5 Statistics

All data is represented by scatter dot plots constructed in GraphPad Prism v8.1.1 (GraphPad, La Jolla, California), with individual data points representing one biological replicate. The mean of each group is provided with a horizontal bar and error bars represent one SD. All statistics were performed in GraphPad Prism v8.1.1 (GraphPad). All data were considered significant at *P ≤* .05. In Aim 1 (ie, in vivo experiment), data was first evaluated with a two‐way ANOVA to determine significance of sex and diet. Two‐tailed independent *t* tests were applied to compare differences due to dietary groups. In the second half of the experiment (ie, ex vivo organ culture) a two‐way ANOVA with post hoc Bonferroni analysis was used to compare differences between culture conditions and RAGE.

## 3 RESULTS

### 3.1 Dietary AGEs led to an increase in collagen fibril disruption and a decrease in the total amount of collagen

Sex had no significant effect for SHG intensity (*P* = .9070), collagen fibril disruption (*P* = .1865), collagen percent area (*P* = .2683), and collagen damage (*P* = .1310); therefore both sexes were pooled for further evaluation.

**FIGURE 2:**
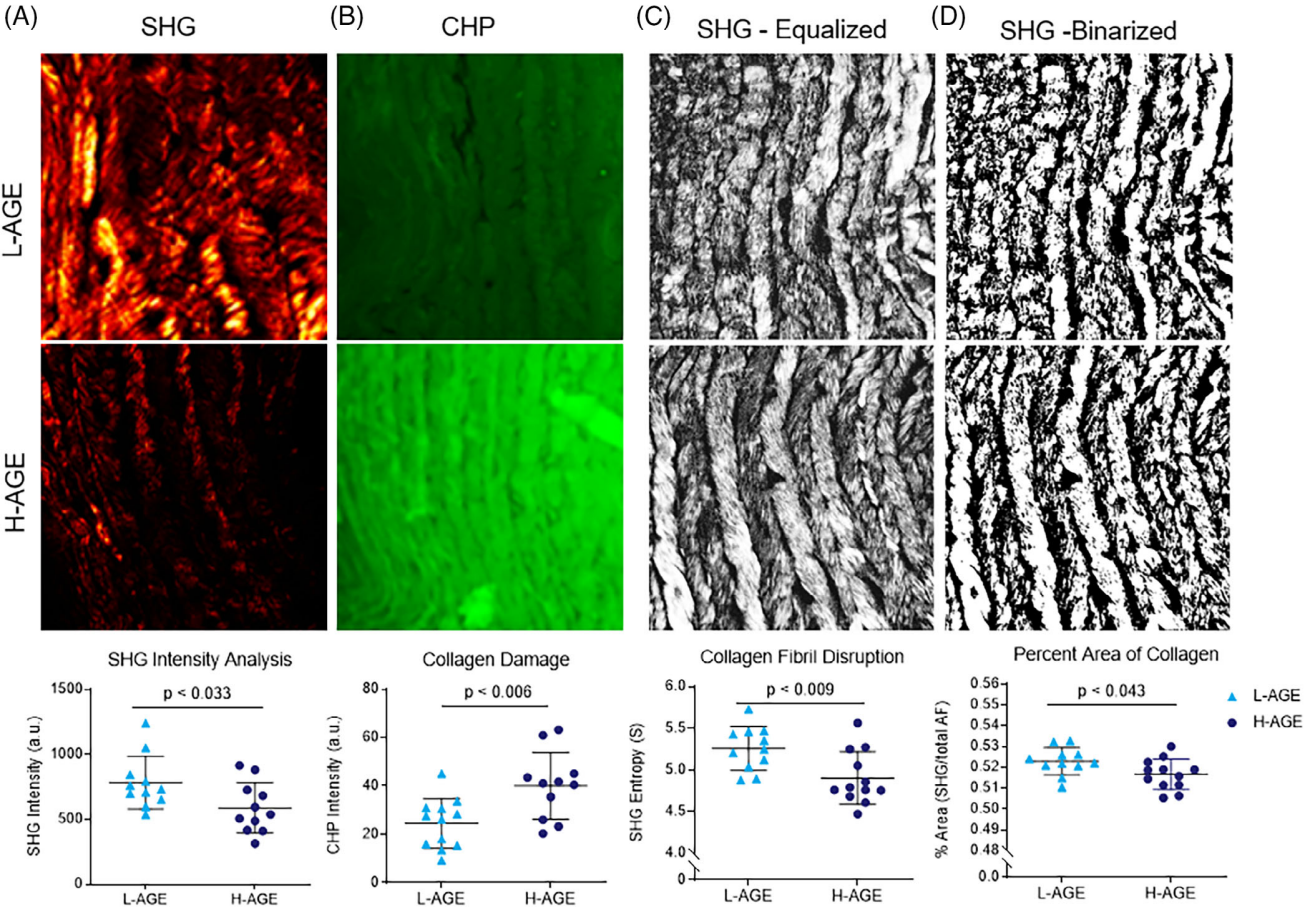
H‐AGE diet caused increased collagen damage, increased fibril disruption, and decreased total collagen amount. A, SHG images of the central anterior AF region were quantified for SHG intensity. (n = 10, n = 11). B, CHP images of the anterior AF indicated collagen damage in the central anterior AF (n = 12, n = 11). SHG images were C, equalized and then D, segmented to perform morphometric analysis and entropy and percent area was quantified as a measure of collagen fibril disruption and total amount of collagen respectively (n = 10, n = 11). *P* values represent two‐tailed *t* test comparing the L‐AGE and H‐AGE groups. AF, annulus fibrosus; AGEs, Advanced glycation end products; CHP, collagen‐hybridizing peptide; SHG, second harmonic generation

In response to a H‐AGE diet, IVDs exhibited significantly decreased mean SHG intensity (percent difference − 25.8%; *P* < .033; Figure 2A). CHP analyzes identified significantly increased signal intensity demonstrating increased collagen damage (percent difference 63.5%; *P* < .006; Figure 2B).^15^ Texture analysis of the SHG images demonstrated significantly decreased SHG entropy in the H‐AGE group, indicating fibril level disruption of the collagen ECM in the anterior AF (percent difference − 6.76%; *P* < .009; Figure 2C). Finally, the percent area of SHG signal was significantly but slightly decreased in the H‐AGE group (percent difference − 1.19%, *P* < .043), suggesting a subtle decrease in the total amount of collagen in the anterior AF (Figure 2D).

### 3.2 RAGE dependent increase in collagen damage at multiple hierarchical levels in an IVD ex vivo organ culture model

**FIGURE 3:**
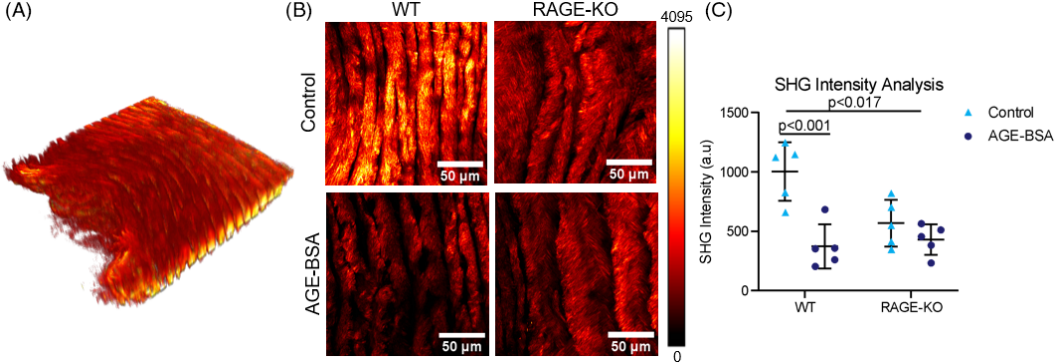
AGE‐BSA caused reduced SHG intensity in WT but not RAGE‐KO mice. A, SHG 3D reconstruction of ex vivo organ culture of WT and RAGE‐KO IVDs to observe collagen microstructure with minimal processing of tissue. B, A maximum intensity z‐projection of the central anterior AF was created for image analysis. C, SHG intensity was quantified and two‐way ANOVA with Bonferroni post hoc (n = 5). *P <* .017 represents contribution of RAGE‐KO genotype to decreased SHG Intensity, while *P <* .001 compares SHG Intensity of WT IVDs cultured in AGE‐BSA supplemented media. AF, annulus fibrosus; AGEs, Advanced glycation end products; IVD, intervertebral disk; RAGE, receptor for AGEs; RAGE‐KO, RAGE‐knockout; SHG, second harmonic generation; WT, wildtype

**FIGURE 4:**
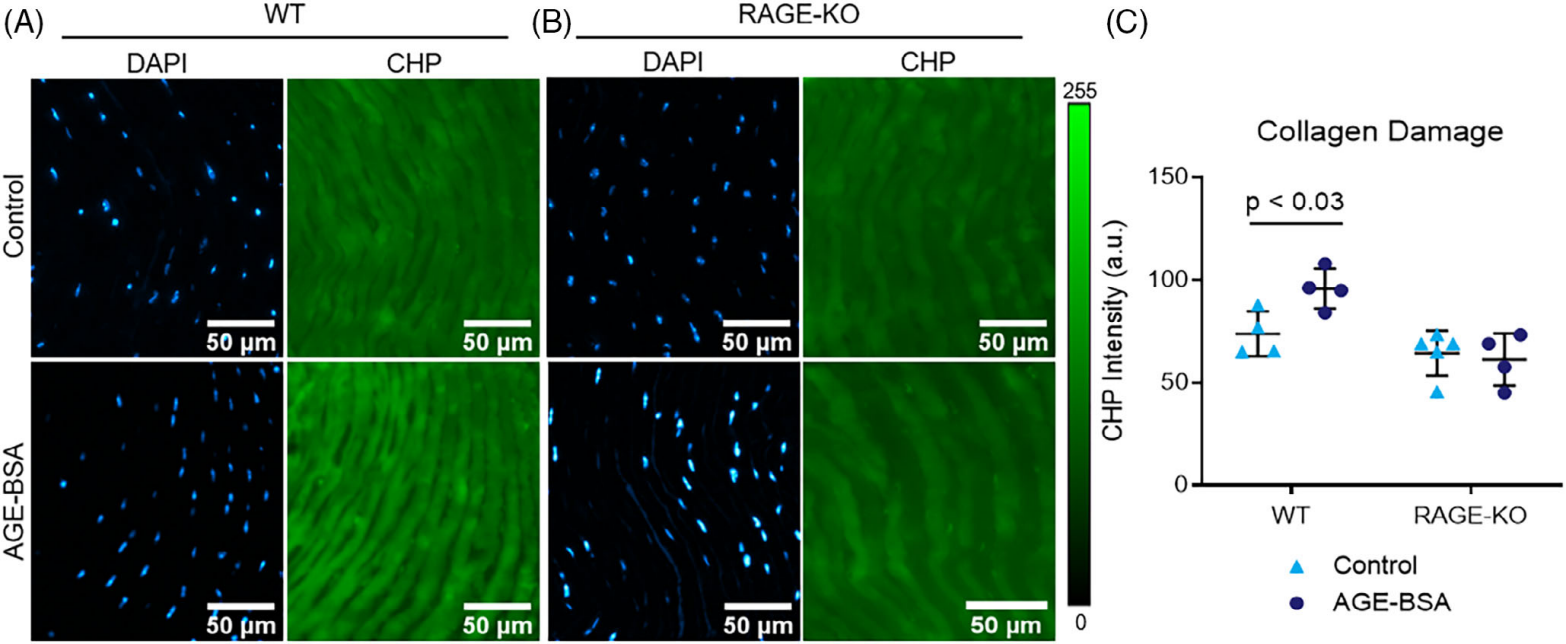
AGE‐BSA caused collagen damage in WT but not RAGE‐KO mice. CHP analysis was performed on A, WT and B, RAGE‐KO IVDs cultured in AGE‐BSA or BSA control media. Blue indicates DAPI labeled cell nuclei, green represents fluorescein labeled CHP molecules incorporated into damaged collagen. Two‐way ANOVA with Bonferroni multiple comparison *P* value is presented (n = 5). AGEs, Advanced glycation end products; CHP, collagen‐hybridizing peptide; IVD, intervertebral disk; RAGE, receptor for AGEs; RAGE‐KO, RAGE‐knockout; WT, wildtype

In situ SHG 3D reconstruction and maximum intensity z‐projections were used to evaluate the overall tissue morphology of the outer AF (Figure 3A,B). Culture with AGE‐BSA significantly decreased SHG intensity of the anterior AF in the WT IVDs (percent difference − 62.8%; *P* < .001; Figure 3C). The key result is that AGE‐BSA caused a significant reduction in SHG intensity in WT while AGE‐BSA did not cause a significant reduction in SHG intensity for RAGE‐KO. It is also notable that SHG intensity values for WT and RAGE‐KO had different baseline levels, which were significantly different when comparing between Control WT and AGE‐BSA RAGE‐KO (*P* < .017). To assess the role of RAGE in changes to collagen we evaluated RAGE‐KO and determined that RAGE‐KO was protective against this loss of SHG intensity (*P* > .999). We therefore directly measured changes in collagen degradation using CHP analysis, and determined that AGE‐BSA caused significantly increased the binding of CHP to collagen indicating greater collagen damage (percent difference 29.8%, *P* < .030; Figure 4A). RAGE‐KO IVDs were not affected by AGE‐BSA media (*P* > .999; Figure 4B), supporting the concept that AGEs caused RAGE‐dependent molecular degradation of collagen (Figure 4C).

**FIGURE 5:**
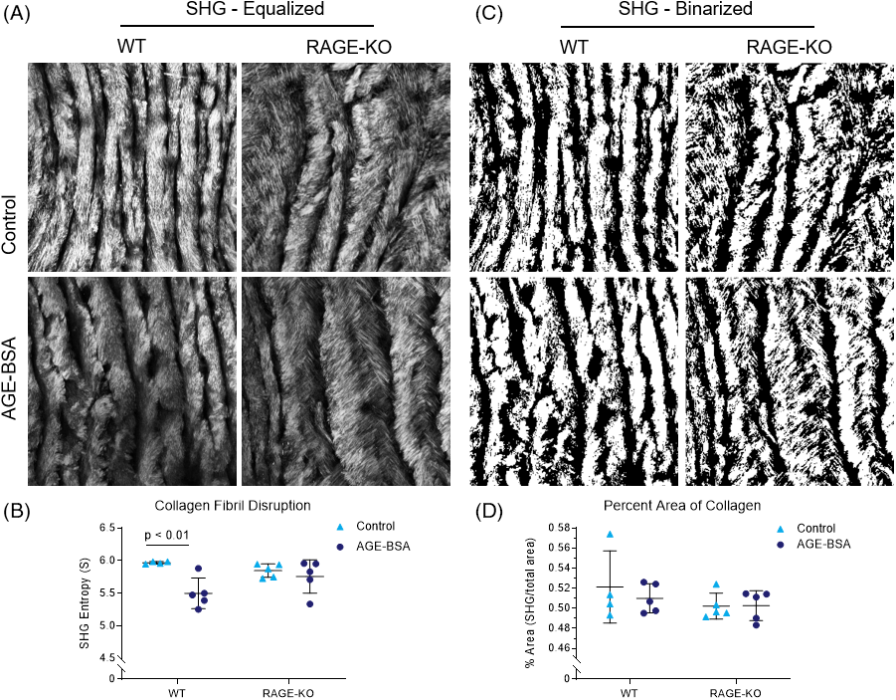
AGE‐BSA caused disrupted collagen fibril structure in WT but not RAGE‐KO mice but no changes in percent area collagen. Morphometric analysis of A, Intensity equalized SHG images and B, Binarized in situ SHG images of mouse central anterior AF were used to quantify, C, SHG entropy as a measure of fibril disruption, and D, Percent area of SHG signal as a measure of total amount of collagen. Two‐way ANOVA with Bonferroni multiple comparison *P* value is presented (n = 5). AF, annulus fibrosus; AGEs, Advanced glycation end products; RAGE‐KO, RAGE‐knockout; SHG, second harmonic generation; WT, wildtype

To determine if the damage to the molecular structure of collagen was linked to the higher order levels of collagen fibril structure, we evaluated SHG entropy. Texture analysis showed a significantly decreased SHG Entropy of the WT IVDs cultured in AGE‐BSA (percent difference − 7.86%; *P* < .012; Figure 5A). SHG Entropy was not affected by AGE‐BSA media in the RAGE‐KO groups (*P* > .999; Figure 5B). Finally, AGE‐BSA media caused no changes were detected in the amount of collagen (*P* > .576; Figure 5C,D).

## 4 DISCUSSION

Developing improved methods to assess early degenerative changes to IVD collagen can advance our understanding of IVD degeneration mechanisms and identify opportunities for intervention. This study evaluated the role of AGEs and RAGE in driving early IVD degeneration processes in mice. We characterized IVD collagen by applying multiphoton microscopy with SHG on IVD thin sections and in situ explants along with CHP and image processing analyzes. This in vivo dietary model was previously shown to increase IVD AGE accumulation without systemic obesity or diabetes.^15^ The current study identified AF collagen damage at the molecular and fibrillar levels, and collagen loss. Organ culture results demonstrated that these collagen molecular and fibrillar changes were IVD specific, not requiring the presence of an immune system or other factors present in the in vivo model. Furthermore, since RAGE‐KO was protective of most collagen changes, we conclude that the observed collagen structural damage was RAGE dependent and suggested that these effects are due to catabolic processes in addition to crosslinking.

**FIGURE 6:**
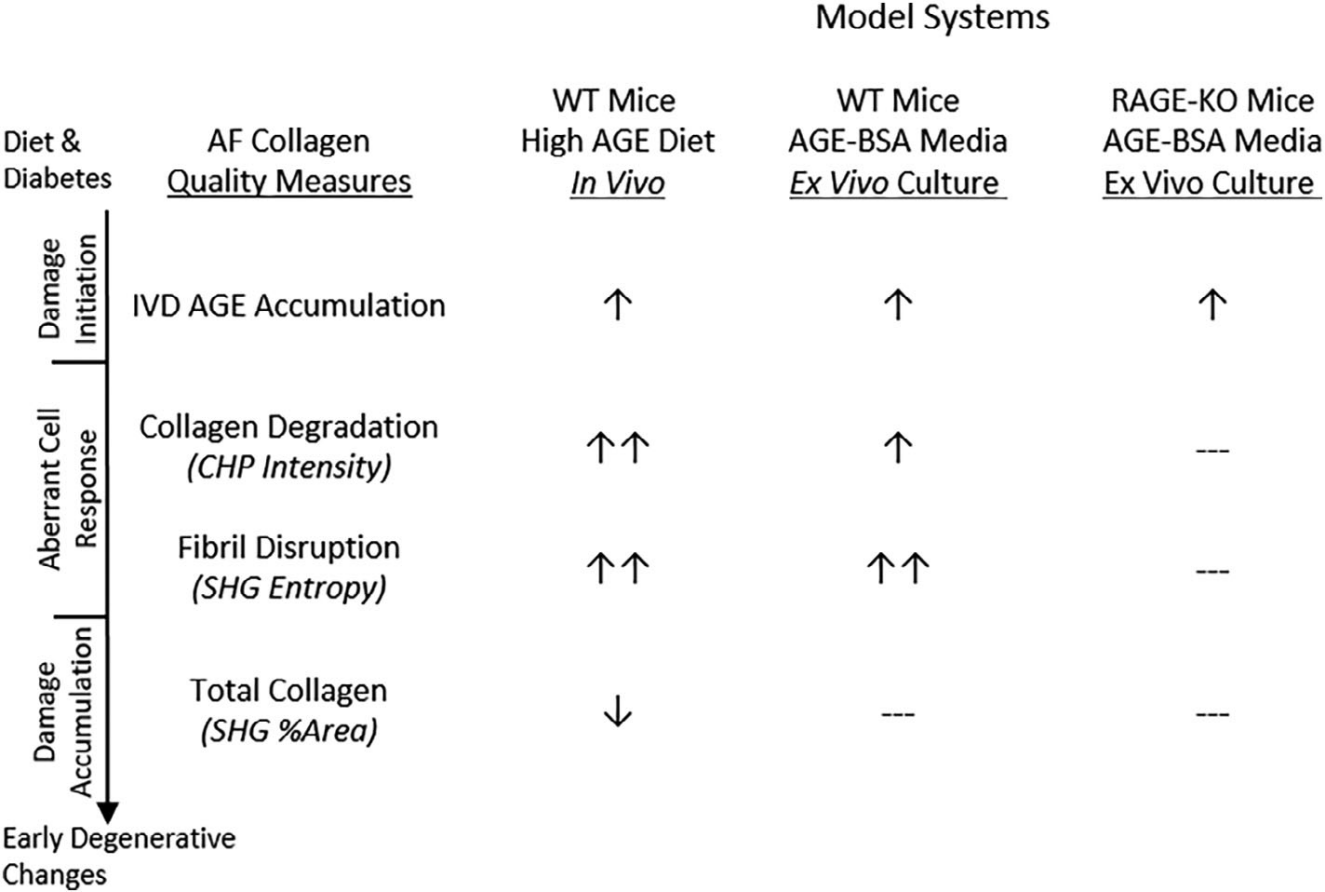
Summary of in vivo dietary model and ex vivo organ culture results for WT and RAGE‐KO mice. Results show a RAGE‐dependent link between AGEs and collagen disruption at the molecular and fibril levels. AF, annulus fibrosus; AGEs, Advanced glycation end products; CHP, collagen‐hybridizing peptide; RAGE, receptor for AGEs; RAGE‐KO, RAGE‐knockout; SHG, second harmonic generation; WT, wildtype

Dietary AGEs as well as diabetes in mice can cause AGE accumulation in IVDs and endplate with a shift toward catabolism and early degenerative changes.^13^, ^43^ AGE accumulation in IVD in rodent models of diabetes and H‐AGE diet was further demonstrated to result in IVD AGE accumulation as well as stiffening providing further evidence of crosslinking from glycation.^14^, ^15^ This study builds on that knowledge and supports the concept of glycoxidation by demonstrating H‐AGE diet and AGE supplemented IVD organ culture causes increased catabolism with AF collagen degradation, fibril disruption and loss of total collagen that is RAGE dependent (Figure 6). This study, together with the literature therefore indicates that IVD AGE exposure, as might be expected from H‐AGE diets or diabetes, can play a causal role in early IVD degeneration. It is interesting to consider that severe AF damage and degeneration, as can occur in mice from severe puncture injuries, can result in reduced IVD stiffness from severe AF disruption in contrast to the literature showing IVD AGE accumulation can result in increased IVD stiffening.^44^, ^45^, ^46^ We therefore believe the AF damage is relatively mild from this dietary intervention and organ culture model and did not progress to the point where collagen damage dominates crosslinking to reduce stiffness. Nevertheless, the AF damage observed was detectable and likely to accumulate and predispose to accelerated IVD degeneration.

**FIGURE 7:**
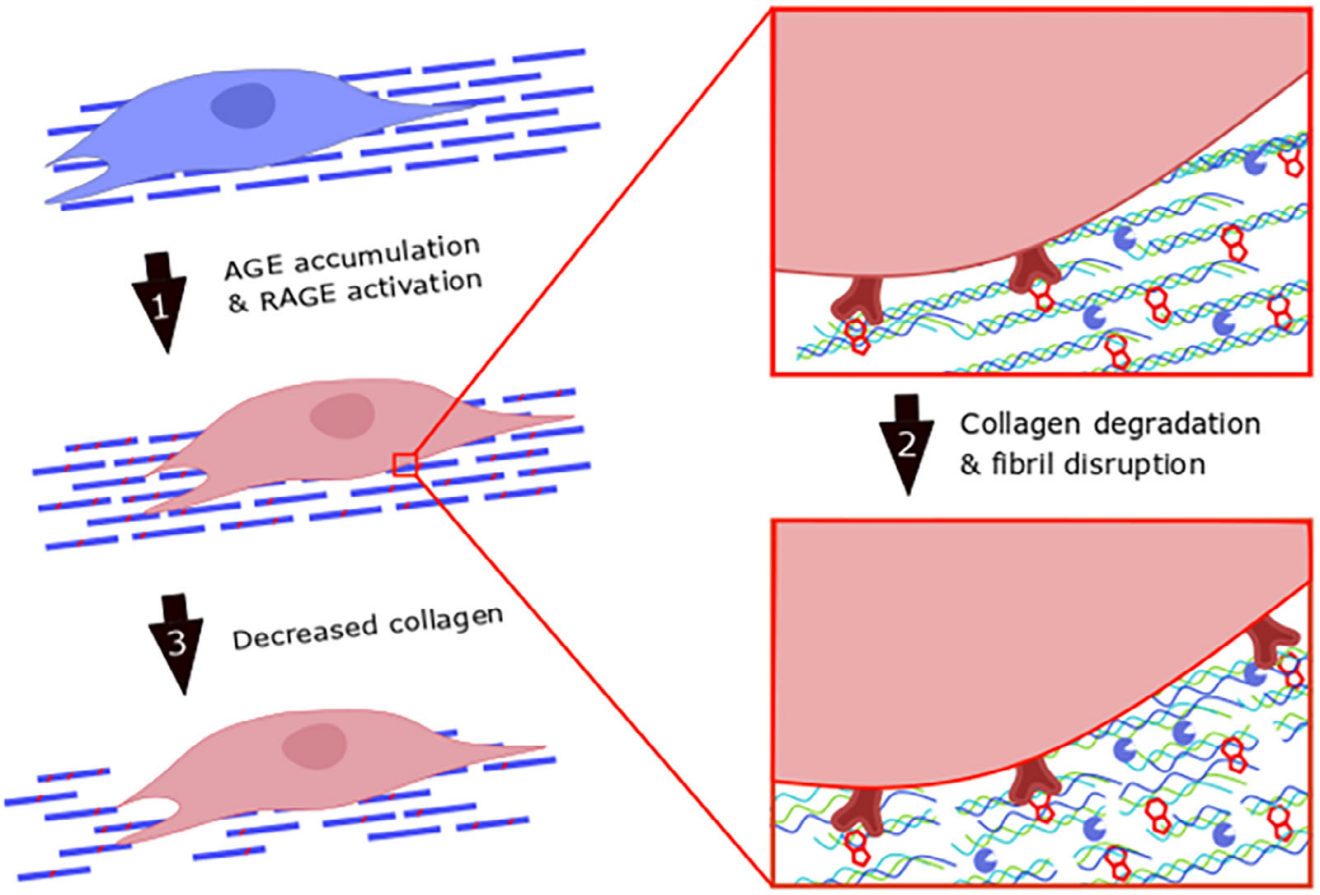
Conceptual model for AGE modifications to reduce collagen quality and quantity. (1) AGEs accumulate in the IVD matrix to cross‐link collagen molecules and activate the RAGE pathway. (2) AGEs cause RAGE‐dependent increased catabolism with collagen degradation and fibril disruption. (3) Chronic collagen degradation results in eventual decrease in collagen. AGEs, Advanced glycation end products; IVD, intervertebral disk; RAGE, receptor for AGEs

AGEs have been previously shown to stimulate expression of inflammatory catabolic enzymes MMP1, MMP3, and MMP9 in IVD cells.^47^, ^48^, ^49^ AGEs also cause oxidative stress and proinflammatory cytokine production,^12^ which are known drivers of IVD degeneration.^50^, ^51^, ^52^ Increased carbonylation (a form of glycation) can also directly cause structural disruption to molecular collagen, increasing susceptibility to degradation by MMPs.^53^ However, in the absence of initial molecular damage to collagen, glycation formation has also been shown to protect collagen from degradation via MMPs.^54^ Since IVD collagen degradation and fiber disruption occurred both in vivo as well as in an ex vivo model, we conclude that these crosslinking and catabolic processes occurred in an IVD specific way, due in part to IVD cells in these living models. Furthermore, RAGE‐KO protected IVDs from collagen damage, which supports that this is a RAGE‐dependent process. This study demonstrated a direct role of AGE formation and RAGE in causing IVD cells to adopt this degenerative phenotype from matrix catabolism (Figure 7).

This study developed in situ SHG imaging methods as a step toward a vision of developing noncontacting and nondestructive manner of precisely measuring collagen organization and quantity without the need for histological processing. Aim 1 controlled for histological processing methods by treating experimental and control samples similarly, yet histological methods can influence SHG measurements motivating our development of in situ SHG analyzes in Aim 2. In situ SHG analyzes controlled for the depth of laser penetration across tissues and compared intensity of emitted signals by creating a mid‐sagittal cut that was applied to expose a single cross‐section of AF for imaging. Therefore, the in situ imaging techniques used here remain an endpoint measurement. While we succeeded in imaging live IVD tissues with minimal processing, additional work would be required to develop methods of assessment imaging from the outer AF surfaces, which would be required for a true nondestructive test enabling repeated time point measurements in the adult mice IVDs in vivo. Repeated measurements with time would allow researchers to assess subtle changes to collagen as well as perform intravital video microscopy of labeled cells within the IVD and may eventually enable application of in vivo SHG imaging methods. Although in vivo intravital video imaging of the IVD has yet to be reported, in situ 3‐dimensional intravital imaging of the IVD was reported previously to study the transition of NC cells to NP cells,^55^, ^56^, ^57^ further suggesting in vivo SHG analyzes are possible.

Texture analysis is a group of second order parameters that consider the intensity of individual pixels with respect to the pixels in its nearest neighborhood. Together this group of parameters is known as the gray level co‐occurrence matrix (GLCM) which includes the entropy parameter included in this work. The other GLCM parameters are energy, inertia, inverse difference moment, and correlation.^58^ While each parameter is known to give unique information about SHG texture, we used entropy in this study to assess collagen quality since it has been used to assess collagenous changes at the fibril level, it was sensitive during initial analysis of these parameters, and it is a readily available calculation in the Matlab image analysis toolbox.^28^, ^29^ Previous SHG image analysis techniques in the IVD have focused on the orientation of collagen fibrils throughout the AF under hyper‐physiological loading.^19^, ^20^ This work represents the first time that texture analysis was utilized to characterize fibril disruption in the AF. A potential avenue of future work is the utilization of texture analysis on the spatial distribution of fibril orientations, which may aid in further characterizing early degenerative changes that are yet too subtle to be measured through means such as birefringence distributions or semiquantitative scoring.

In vivo and ex vivo organ culture results yielded similar findings with more severe alterations occurring in vivo. The degree of collagen loss observed in vivo was modest, and was not observed in the ex vivo culture. We attribute the greater severity of collagen damage in vivo to the substantially longer duration, which allowed damage to progress with subtle loss of collagen content. However, it is possible that these differences are also attributable to the additional systemic effects or biomechanical factors in vivo. Furthermore, potential differences in sensitivity are possible across thin section imaging vs in situ SHG imaging methods. For CHP measurements, the imaging parameters were different between the in vivo and ex vivo experiments in order to prevent oversaturation, and therefore it is best to compare CHP intensity values as relative within each experiment and not an absolute value. Nevertheless, even with some differences across model systems, many similarities were observed strengthening our confidence in our conclusions, while also showing that organ culture studies can be used to identify a mechanistic role for RAGE. In addition, we observed a significant difference in SHG intensity in ex vivo control culture conditions when comparing WT and RAGE‐KO IVDs (Figure 3C). This baseline difference suggests a role for RAGE in the deposition and/or maturation of collagen in the AF, the implications of which remain unknown. Additional studies are required to investigate the role of RAGE in mediating the baseline structure of IVD collagen. Both sexes were assessed in Aim 1 and only males in Aim 2. Sex did not significantly contribute to the AF changes based on this statistical analysis. Since no sex differences were identified in our Aim 1 studies, we used a single sex for organ culture studies to reduce potential variability due to size and other factors. While this study is limited since we did not directly test for effects of AGEs on female IVDs in organ culture, we believe it is likely that females would also exhibit effects of AGES since female mice were reported to exhibit greater sensitivity than males in other spinal changes from dietary AGEs in our prior study.^15^ Interestingly, this in vivo cohort showed sex‐dependent effects on spine the biomechanical function. Therefore this study helps to clarify that the local effects of AGEs on AF collagen are likely not sex‐dependent, while other spinal tissues (eg, vertebrae) and properties do have sex‐dependent effects of AGEs.

In conclusion, there is a causal relationship between AGEs, RAGE, and collagen degradation in the mice IVDs. The importance of the AGE‐RAGE axis in IVD degeneration shown in this study may inform potential therapies for IVD degeneration in conditions where AGE accumulation is implicated, such as aging and diabetes. This study also demonstrated a method of in situ multiphoton microscopy in the mice IVD with quantifiable image analysis techniques for assessment of collagen quality. Collagen damage was also demonstrated with the novel CHP with direct imaging. These multiphoton microscopy and image analysis methods may be used in the assessment of collagen quality to further understand several degenerative cascades in the IVD.

## CONFLICT OF INTEREST

The authors declare no potential conflict of interest.

## AUTHOR CONTRIBUTIONS

Robert C. Hoy performed in vivo dietary mouse model and organ culture experiments, analyzed data, interpreted results, and wrote the first draft of the manuscript. Danielle N. D'Erminio performed organ culture experiments, analyzed data, interpreted results, and contributed to manuscript writing. Divya Krishnamoorthy performed in vivo dietary mouse model experiment, analyzed data, and interpreted results. Devorah M. Natelson performed in vivo dietary mouse model experiment, analyzed data, and interpreted results. Damien M. Laudier performed CHP testing and interpreted results. Svenja Illien‐Jünger contributed to experimental design and interpretation of results. James C. Iatridis initiated the project, contributed to experimental design, interpretation of results, and manuscript writing. All authors reviewed the manuscript.

## Supporting information


**Supplementary Figure 1** Cell viability was maintained in ex vivo organ culture experiments. Cell viability was measured using double staining of MTT with DAPI to validate that cells remained alive and metabolically active in the organ culture system. A single motion segment from each culture was measured. Live cells were confirmed by robust MTT staining (black staining, left image) across cells in all regions of the IVD. DAPI staining (bright blue, right image) stained all cells, so that we could confirm that MTT staining was aligned closely with cellularity

**Supplementary Figure 2** Collagen damage caused decreased SHG intensity and increased CHP intensity. Heat treatment was used to create positive controls for SHG and CHP analyzes. Heat‐treated samples demonstrated substantial reductions in mean SHG intensity. Heat treatment substantially increased CHP signal intensity as compared to the untreated sample, which had little CHP staining intensity
