# Supplementary figures and images for: Advanced glycation end products cause RAGE‐dependent annulus fibrosus collagen disruption and loss identified using in situ second harmonic generation imaging in mice intervertebral disk in vivo and in organ culture models
Source: JOR Spine. 2020 Sep 21;3(4):e1126. doi: 10.1002/jsp2.1126 (PMC7770195; doi:10.1002/jsp2.1126)

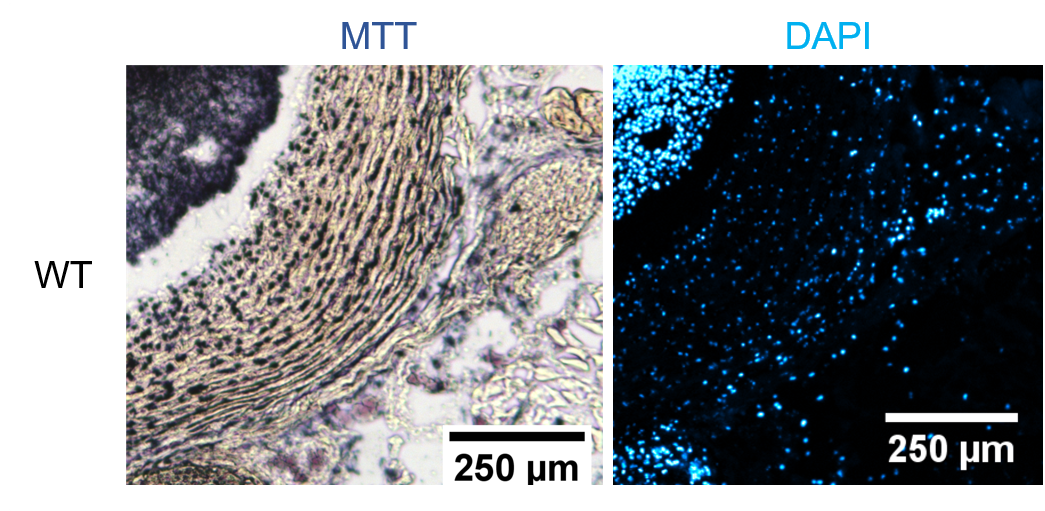

Supplement: Supplementary file 1 — Supplementary Figure 1 Cell viability was maintained in ex vivo organ culture experiments. Cell viability was measured using double staining of MTT with DAPI to validate that cells remained alive and metabolically active in the organ culture system. A single motion segment from each culture was measured. Live cells were confirmed by robust MTT staining (black staining, left image) across cells in all regions of the IVD. DAPI staining (bright blue, right image) stained all cells, so that we could confirm that MTT staining was aligned closely with cellularity [file JSP2-3-e1126-s001.tif]

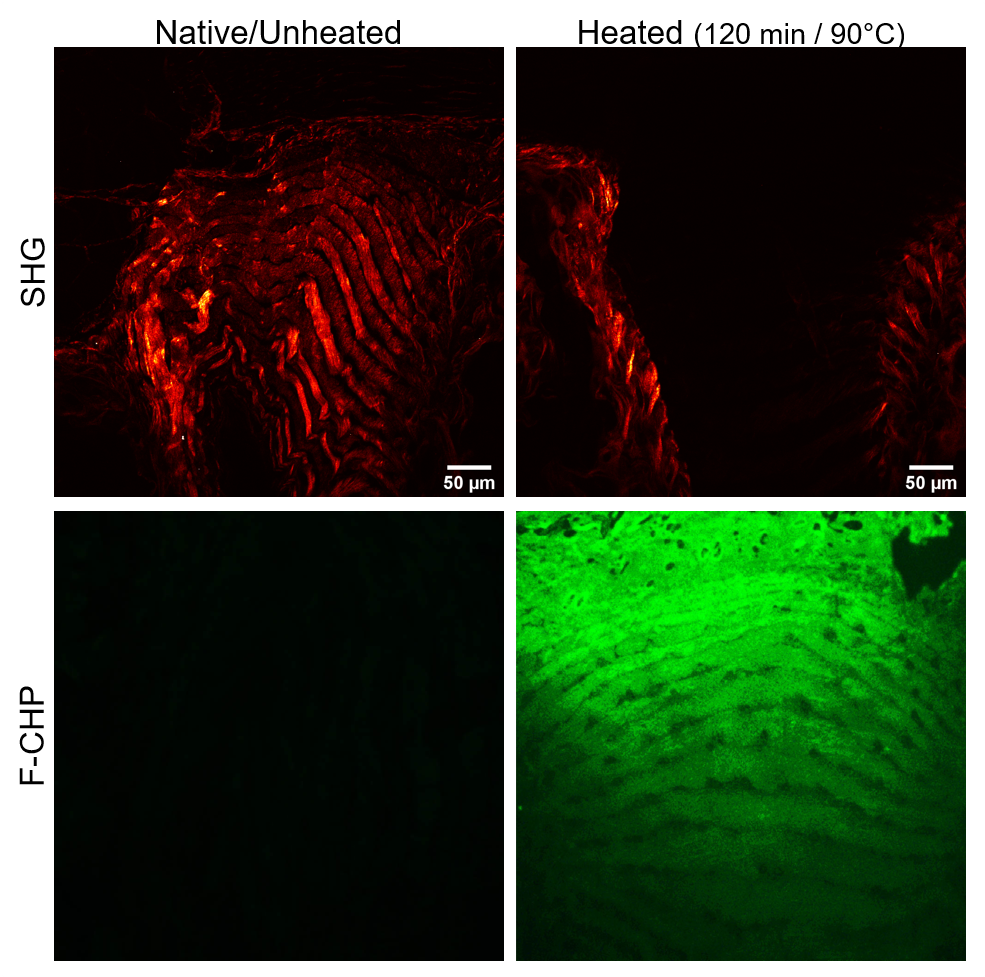

Supplement: Supplementary file 2 — Supplementary Figure 2 Collagen damage caused decreased SHG intensity and increased CHP intensity. Heat treatment was used to create positive controls for SHG and CHP analyzes. Heat‐treated samples demonstrated substantial reductions in mean SHG intensity. Heat treatment substantially increased CHP signal intensity as compared to the untreated sample, which had little CHP staining intensity [file JSP2-3-e1126-s002.tif]
